# QX-type infectious bronchitis virus infection in roosters can seriously injure the reproductive system and cause sex hormone secretion disorder

**DOI:** 10.1080/21505594.2023.2185380

**Published:** 2023-03-08

**Authors:** Kun Yan, Xiuling Wang, Zifan Liu, Zongyi Bo, Chengcheng Zhang, Mengjiao Guo, Xiaorong Zhang, Yantao Wu

**Affiliations:** a Jiangsu Co-Innovation Center for the Prevention and Control of Animal Infectious Disease and Zoonoses, College of Veterinary Medicine, Yangzhou University, Yangzhou, Jiangsu, China; b International Joint Research Laboratory of Agricultural and Agri-product Safety, the Ministry of Education of China, Yangzhou University, Yangzhou, Jiangsu, China

**Keywords:** Infectious bronchitis virus, QX, pathogenicity, reproductive, rooster, hormone

## Abstract

Since its discovery, QX-type avian infectious bronchitis virus (IBV) has rapidly spread worldwide and become the most prevalent dominant genotype in Asia and Europe. Currently, although the pathogenicity of QX-type IBV in the reproductive system of hens is widely and deeply understood, its pathogenicity in the reproductive system of roosters remains largely unknown. In this study, 30-week-old specific pathogen-free (SPF) roosters were used to investigate the pathogenicity of QX-type IBV in the reproductive system after infection. The results showed that QX-type IBV infection caused abnormal testicular morphology, moderate atrophy and obvious dilatation of seminiferous tubules, and produced intense inflammation and obvious pathological injuries in the ductus deferens of infected chickens. Immunohistochemistry results showed that QX-type IBV can replicate in spermatogenic cells at various stages and in the mucous layer of the ductus deferens. Further studies showed that QX-type IBV infection affects plasma levels of testosterone, luteinizing hormone, and follicle-stimulating hormone as well as causes changes in transcription levels of their receptors in the testis. Furthermore, the transcription levels of StAR, P450scc, 3βHSD and 17βHSD4 also changed during testosterone synthesis after QX-type IBV infection, indicating that the virus can directly affect steroidogenesis. Finally, we found that QX-type IBV infection leads to extensive germ cell apoptosis in the testis. Collectively, our results suggest that QX-type IBV replicates in the testis and ductus deferens, causing severe tissue damage and disruption of reproductive hormone secretion. These adverse events eventually lead to mass germ cell apoptosis in the testis, affecting the reproductive function of roosters.

## Introduction

Avian infectious bronchitis (IB) is an acute, highly infectious disease of chickens caused by infectious bronchitis virus (IBV), which belongs to the *Coronaviridae* family [1]. IBV mainly causes respiratory symptoms in chicks and adult chickens and can reduce daily weight gain and the feed conversion ratio in broilers. Some strains cause renal pathogenicity, which can trigger the appearance of urate deposits resulting in an appearance known as “spot-kidney,” nephritis, and even death from renal failure [2]. IBV infection can lead to a decrease in egg production and quality, such as soft-shell eggs, deformed eggs, and thin egg white in laying hens [3]. After infection, the egg production of immunized laying hens generally recovers but not to normal levels. Coinfection of IBV with other pathogens (e.g. H9N2 avian influenza and *Mycoplasma synoviae*) can aggravate the disease [4,5].

IBV has many serotypes and genotypes, and different types of strains may differ significantly in pathogenicity and tissue tropism. The QX-type (also known as LX4 in China) was first discovered in the late 1990s [6]. According to the latest Valastro V typing system, the QX strain was classified into the GI-19 lineage of the GI genotype, which has spread rapidly worldwide since its discovery [7]. It remains the most prevalent strain in many regions of Europe and Asia, including China [8,9]. In addition to its strong respiratory and renal pathogenicity, QX-type IBV has attracted much attention for its pathogenicity in the reproductive system of laying hens [10–12]. It can lead to abnormal development of the oviduct in chicks, and the specific manifestations are dysplasia or cysts with serum-like fluid accumulation [12,13]. This injury to the oviduct is permanent, which leads to the inability to lay eggs normally due to the undeveloped oviduct or the presence of cystic oviduct, although the ovary can ovulate normally during the laying period [14]. A condition called “false layer” syndrome develops in this condition. If infection occurs during the laying period, marked expansion of tubular glands, interstitial dilatation, and lymphocytic infiltration can be observed [15]. Although there is a wide consensus regarding the abnormality of the reproductive system caused by IBV infection in hens, research on the effect of IBV infection on the reproductive system of roosters is very limited, and the few studies have mainly reported manifestations such as testicular atrophy, orchitis, and epididymal lithiasis [16,17]. Another study showed that individual IBV strains could replicate in the testis under laboratory conditions, but no obvious pathological changes were observed, and the virus carried in semen could be transmitted to hens by mating [18]. Recently, due to the emergence of coronavirus disease 2019 (COVID-19), some studies have confirmed the pathogenicity of severe acute respiratory syndrome coronavirus 2 (SARS-CoV-2) in the human reproductive system, especially in the male reproductive system, which causes long-term and irreversible injury [19,20]. Thus, there is a need for focus on the impacts of IBV, which is also in the *Coronaviridae* family, on the reproductive system of roosters.

In this study, QX-type IBV, which is highly pathogenic to the reproductive system of hens, was used to infect 30-week-old SPF roosters as experimental subjects, and the pathogenicity and potential impacts of QX-type IBV on the rooster reproductive system were evaluated from the aspects of gross lesions, viral load, plasma reproductive hormone levels and cellular apoptosis.

## Materials and methods

### Virus

The QX-type velogenic IBV strain CK/CH/JS/2010/12 (abbreviation: QXL) was originally isolated in 2010 from the trachea and kidney of chickens that exhibited respiratory symptoms and death (GenBank accession NO. JQ900122.1). The 50% chicken embryo infectious dose (EID_50_) of the QXL strain was calculated using the Reed and Muench method as described previously [21].

### Animals and ethics statement

One-day-old SPF white Leghorn chickens were purchased from Jinan Sipai Furui Livestock Technology Co., Ltd. (Shandong, China) and kept in negative-pressure isolators in the secondary biosafety barrier area until 30 weeks of age.

All the animal experimental procedures were performed in accordance with the ARRIVE Guidelines 2.0. All experimental protocols applied in this study were approved by the Animal Welfare and Ethical Review Committee of Yangzhou University (202103403).

### Experimental design

All the birds were randomly divided into two groups with 10 birds in each group and housed in negative-pressure isolators in the secondary biosafety barrier area. All animals were provided ad libitum access to food and water during the experiment. At the age of 30 weeks, animals in the challenge group were inoculated with 200 μL of allantoic fluid containing 10^5^ EID_50_ of the QXL strain via the oculonasal route. Birds in the control group were administered 200 μL of phosphate-buffered saline (PBS) via the oculonasal route. At 4 and 8 days post-infection (dpi), five birds from each group were randomly selected for blood and tissue sample collection. Blood samples were collected from the wing vein, gently added to tubes coated with heparin sodium, and immediately centrifuged at 3000 × g for 20 min at 4 °C. Plasma samples were obtained and stored at −70 °C. Then, the birds were euthanized by cervical dislocation, body and testicular weights were recorded, and gross lesions of the testis and ductus deferens were observed. The results are presented as the GSI (expressed as the ratio of testicular weight to body weight). Testis and ductus deferens tissue samples were subsequently collected, some of which were immediately fixed in 10% neutral-buffered formalin. The remaining tissue was placed in a prelabeled cryogenic storage tube, immediately flash-frozen in liquid nitrogen for 1 h, and then frozen at −70 °C.

### Histopathology and immunohistochemistry

The fixed tissue samples were processed via the standard histological procedure, embedded in paraffin wax, and cut into 5-μm sections. Then, some sections were stained with haematoxylin and eosin (H&E) for histopathological observation. Some sections were prepared for immunohistochemistry (IHC) to detect the distribution and accumulation of the viral antigen in tissues. Briefly, sections were treated with 3% hydrogen peroxide for 6 min to eliminate the activity of endogenous peroxidase. Sections then underwent the antigen retrieval procedures, followed by blocking with 10% (v/v) goat serum for 30 min. The samples were then sequentially incubated with a mouse anti-IBV N protein monoclonal antibody at 37 °C for 2 h and a biotin labelled goat anti-mouse antibody at 37 °C for 30 min, followed by development with a concentrated SABC-POD (mouse IgG) kit (BOSTER Bioengineering Co., Ltd.). Sections were visualized using a 3,3’-diaminobenzidine (DAB) Chromogenic Substrate Kit (Brown) (BOSTER Bioengineering Co., Ltd.) and counterstained with Mayer’s haematoxylin before mounting the slides with neutral balsam mounting medium. Sections were observed using a Leica DM2000 LED light microscope (Leica, Wetzlar, Germany) and photographed with the accompanying MShot Image Analysis System V1.1.4 (MShot, Guangzhou, China).

### Enzyme-linked immunosorbent assay

Concentrations of plasma luteinizing hormone (LH), follicle-stimulating hormone (FSH), testosterone (T) and oestrogen (E2) were determined by enzyme-linked immunosorbent assay (ELISA) kits (Andy Gene Biotech Co., Ltd.) according to the manufacturer’s protocols. Absorbance was measured at 450 nm using an ELx800 microplate reader (BioTek, USA), and the parameters were set using the accompanying Gen5 software. The sensitivity of the assay was 50 ng/L for LH, 0.5 IU/L for FSH, 0.2 nmol/L for T and 2 ng/L for E2. Concentrations of plasma reproductive hormones were calculated using the standard curve based on the standards provided with the respective kits.

### Real-time quantitative reverse transcription polymerase chain reaction

Total RNA of the testis and ductus deferens (100 mg from each sample) was extracted using an Ultrapure RNA Kit (CoWin Biosciences Co., Ltd.) according to the manufacturer’s instructions, and the concentrations of extracted total RNA were determined using a NanoDrop2000 Ultra microspectrophotometer (Thermo, USA). A total of 5 μg of RNA was then reverse transcribed to cDNA using an EasyScript® Reverse Transcriptase (M-MLV, RNaseH-) Kit (TransGen Biotech Co., Ltd.) and random hexamer primers (GenScript Biotech Co., Ltd.) or oligo (dT)_18_ (Takara Bio). The reaction system was prepared with AceQ® qPCR probe Master Mix (Vazyme Biotech Co., Ltd.) for the detection of viral load by qRT‒PCR, and the primers, probe, and methods of obtaining standard curves were referenced in a previous study [22]. AceQ® qPCR SYBR Green Master Mix (Vazyme Biotech Co., Ltd.) was used to prepare the reaction system for qRT‒PCR to detect the relative expression levels of genes, and the primers designed by Primer Premier 5.0 or based on a previous study are shown in Table 1. The relative mRNA expression levels of genes are presented as fold changes and were calculated using the 2^−ΔΔCT^ method [26]. All the assays were performed in triplicate.

**Table 1.** The primers used for quantitative PCR.

Gene name	Sequence (5’-3’)	Amplicon size (bp)	Accession number	Reference
IL-6	F: ATAAATCCCGATGAAGTGG	145	NM_204628.2	-
	R: TCACGGTCTTCTCCATAAA			
IL-1β	F: CCGCTTCATCTTCTACCGC	135	NM_204524.2	-
	R: TGGTCGGGTTGGTTGGTG			
IFN-γ	F: TAGCTGACGGTGGACCTA	152	NM_205149.1	[23]
	R: CTCAGATATGTGTTTGATGTGCG			
IFN-α	F: ACAGCCAACGCCAAAGCC	115	NM_205427.1	-
	R: AGGTGAAGGTTGCGAGGC			
IFN-β	F: AACACTGGATTGACCGCACA	200	NM_001024836.1	-
	R: GTCCCAGGTACAAGCACTGT			
IFITM3	F: TATGGGAGGACAGCGAAGA	171	NM_001350059.2	-
	R: GGTAGAGGGAAGGAGGAGTG			
OASL	F: GCTGCTGACCATCTACGCCT	156	NM_001397447.1	-
	R: ATACCGACCCACTCATCCTCC			
NOS_2_	F: GCATTCTTATTGGCCCAGGA	66	NM_204961.2	[24]
	R: CATAGAGACGCTGCTGCCAG			
LHR	F: ACTCCTGCGCAAACCCATTC	99	NM_204936.1	[25]
	R: CTCGGCTCTTACAGCAACCT			
AR	F: AGTGCCAGCCCATCTTTCTC	159	NM_001040090.1	[25]
	R: CCTTTGCCCACTTGACGAC			
FSHR	F: AATACCCTGCTAGGACTG	238	NM_205079.1	-
	R: GAATACCCATTGGCTCA			
StAR	F: TTCAGCGAGATGGAGATGTCC	160	NM_204686.2	[25]
	R: GGAACACCTTACCCACGTCC			
P450scc	F: GTTGGGTGTCTACGAGAGCG	126	NM_001001756	[25]
	R: TTGCGGTAGTCACGGTATGC			
3βHSD	F: GGGCAAGACTGAGGTGAAAATC	94	XM_015294370.2	[25]
	R: TGTGTGGATGACGAGCGAG			
17βHSD4	F: CGCTGGAGGAGGTTTGGG	167	NM_204943.1	[25]
	R: TGGGTACTGCTTTCCCTCCA			
PAPSS_2_	F: ATTCCCTGGACGGCGATAA	123	XM_040702863.2	-
	R: GCCCAGCATCAGCAAACAA			
β-actin	F: CGCAAATGCTTCTAAACC	169	L08165.1	-
	R: GACTGCTGCTGACACCTT			

Abbreviations: F, forward; R, reverse; IFITM: interferon-induced transmembrane protein; OASL: 2’-5’-oligoadenylate synthetase like; NOS_2_: nitric oxide synthase 2; LHR: luteinizing hormone receptor; AR: androgen receptor; FSHR: follicle-stimulating hormone receptor; StAR: steroidogenic acute regulatory protein; P450scc (CYP11A1): cytochrome P450 family 11 subfamily A member 1; 3βHSD: 3β-hydroxysteroid dehydrogenase; 17βHSD4: hydroxysteroid 17-beta dehydrogenase 4; PAPSS_2_: 3’-phosphoadenosine 5’-phosphosulfate synthase 2.

### Terminal deoxynucleotidyl transferase dUTP nick-end labeling assay

Apoptotic cells in the testis were identified by a terminal deoxynucleotidyl transferase dUTP nick-end labelling (TUNEL) assay using a One-step TUNEL Apoptosis In Situ Detection Kit (Green FITC Labeled Fluorescence Assay, Universal) (KeyGEN Biotech Co., Ltd.) following the manufacturer’s procedures. Briefly, the prepared dewaxed slides were permeabilized with sodium citrate buffer (pH 6.0, 0.01 mol/L) in a microwave at medium-high heat for 8 min. Sections were washed in PBS (3 × 5 min), and a positive slide was then prepared by covering the slide with a DNase I (50 U/μL) reaction mixture at 37 °C for 30 min. Sections were sequentially incubated with a TdT enzyme reaction mixture at 37 °C for 60 min and streptavidin-fluorescein labelling solution at 37 °C for 30 min in a humidified box, followed by counterstaining with DAPI before mounting the slides with glycerine mounting medium. Sections were immediately examined with an Olympus IX73 fluorescence microscope (Evident (subsidiary corporation of Olympus), Japan) and photographed with the accompanying cellSens software (Olympus, Japan).

### Statistical analysis

All data were statistically analysed via Student’s *t* test using GraphPad Prism 8.3 software (GraphPad, La Jolla, CA, USA). Differences between the challenge and control groups were considered statistically significant when *P* was<0.05.

## Results

### IBV infection causes morphological abnormality and histopathological changes in the testis

**Figure 1.**
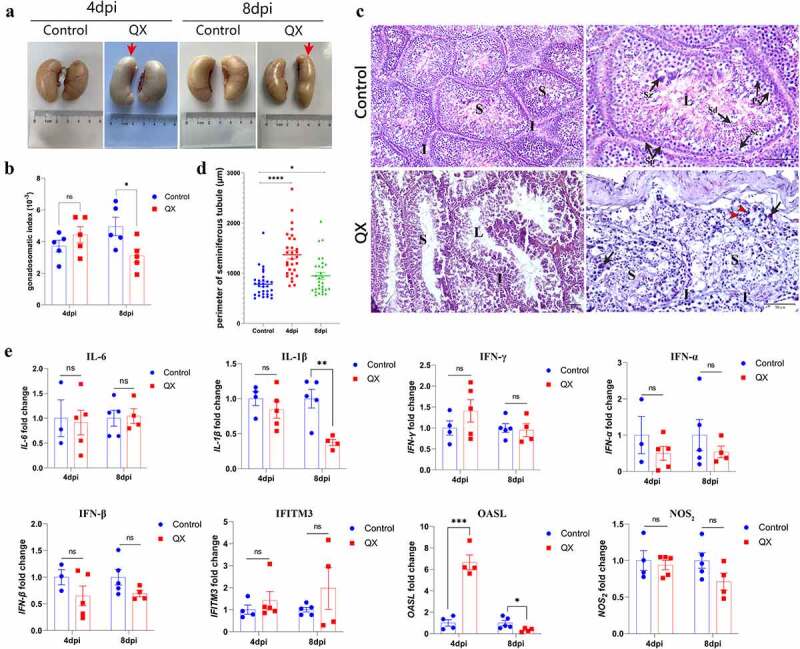
Changes in morphology, histopathology, and the transcription levels of genes involved in the innate immune response in IBV-infected testis. (a) Gross lesions of testis at 4 and 8 dpi. Red arrows indicate morphological abnormalities of the testis. (b) Gonadosomatic index (GSI), expressed as the ratio of testicular weight to body weight (*n* = 5). Data are presented as the mean±sem. *P* < 0.05 (*). (c) Histopathological changes detected in cross sections of testis. Black arrows indicate macrophages, and red arrowheads indicate neutrophils. Abbreviations: S, seminiferous tubule; I, interstitium; L, lumen; Sp, spermatogonium; Ps, primary spermatocyte; Sd, spermatid; Sz, spermatozoon; Sc, Sertoli cell. (d) Perimeter of the seminiferous tubule. The seminiferous tubules were selected randomly, and the data were measured in triplicate (*n* = 30). Data are presented as the mean±sem. *P* < 0.05 (*), *P* < 0.0001 (****). (e) the mRNA expression levels of genes involved in the innate immune response in the testis (*n* = 5). Values are expressed as the mean±sem of the fold change. *P* < 0.05 (*), *P* < 0.01 (**), *P* < 0.001 (***).

To determine the impacts of QX-type IBV on the testis, lesions and histopathological changes were evaluated during necropsy. The normal testis are usually bean-shaped, with slightly different sizes and weights on each side. However, obvious morphological abnormalities were observed in the testis both at 4 and 8 dpi (Figure 1(a)), and the GSI was significantly decreased at 8 dpi (Figure 1(b)). Then, H&E staining was performed for histopathological analysis. The cross section of the normal testis mainly contains seminiferous tubules and interstitium, and the spermatogenic cells at various stages are arranged neatly (Figure 1(c), Control group). Compared with those in the control group, the seminiferous tubules in the challenge group exhibited obvious dilatation, blurred boundaries and disordered spermatogenic cells in each stage, and a small number of macrophages and neutrophils were also observed (Figure 1(c), challenge group). A significant increase of seminiferous tubule perimeters could be observed both at 4 and 8 dpi (Figure 1(d)). Then, the transcription levels of genes involved in the innate immune response were detected to evaluate the degree of inflammation and antiviral immune response. The results showed that the transcription levels of neither proinflammatory cytokines nor interferons (IFNs) were significantly changed, whereas the transcription level of OASL was significantly higher in the challenge group than in the control group at 4 dpi (Figure 1(e)).

### IBV infection causes severe inflammation and histopathological injury in the ductus deferens

**Figure 2.**
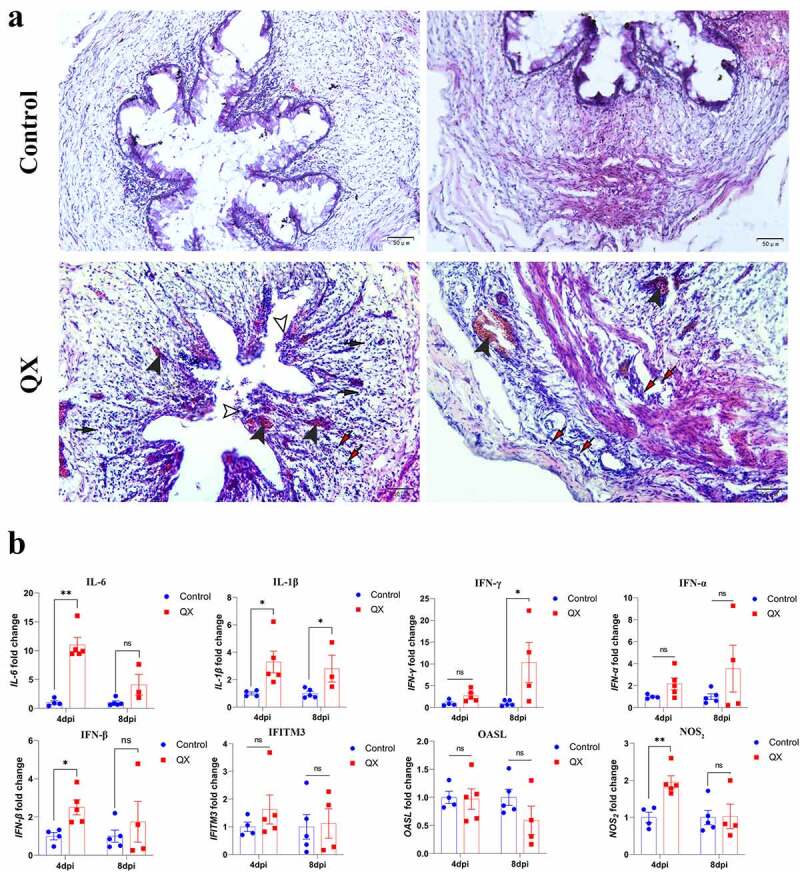
Changes in histopathology and the transcription levels of genes involved in the innate immune response in the IBV-infected ductus deferens. (a) Histopathological changes detected in cross sections of the ductus deferens. White arrowheads: lost cilia and damaged mucosal epithelium; black arrowheads: congestion in the mucous layer; black arrows: diffuse hemorrhagic spots; red arrows: lymphocytes. (b) the mRNA expression levels of genes involved in the innate immune response in the ductus deferens. Values are expressed as the mean±sem of the fold change (*n* = 5). *P* < 0.05 (*), *P* < 0.01 (**).

There were no obvious morphologic lesions in the ductus deferens observed during necropsy. The histopathological changes in the ductus deferens were subsequently evaluated by H&E staining. The cross section of the normal ductus deferens is characterized by mucosal folds covered with a pseudostratified ciliated columnar epithelium (Figure 2(a), control group). Compared to those in the control group, the cilia of pseudostratified ciliated columnar epithelial cells in the challenge group were completely lost, and some of the mucosal epithelial cells were damaged and shed (Figure 2(a), challenge group). Moreover, extensive congestion and haemorrhagic spots were observed in the lamina propria, and lymphocytes were infiltrated into connective tissue and smooth muscle layers (Figure 2(a), challenge group). Similarly, the results showed that the transcription levels of proinflammatory cytokines (IL-6, IL-1β, IFN-γ), NOS_2_ and IFN-β in the challenge group were significantly higher than those in the control group, whereas the transcription levels of IFITM3 and OASL did not significantly change (Figure 2(b)).

### Distribution and quantification of IBV in the testis and ductus deferens

**Figure 3.**
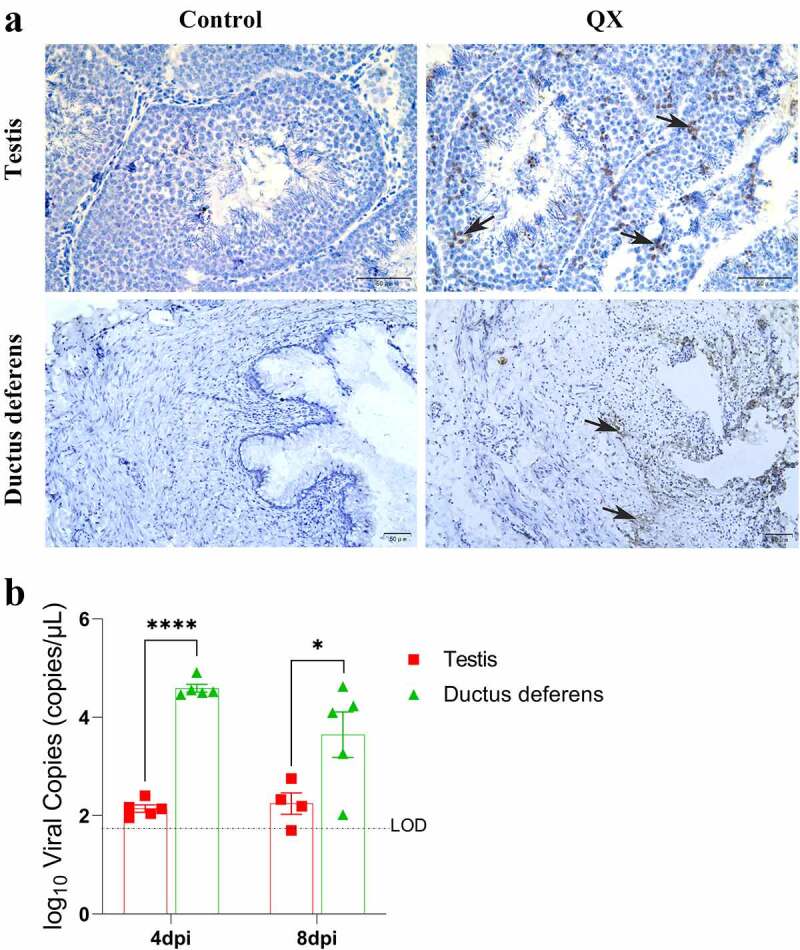
Detection of the viral antigen distribution and viral loads in the testis and ductus deferens by IHC staining and qRT‒PCR. (a) Results of IHC staining. Black arrows indicate antigen deposition. (b) Quantification of viral RNA. The dotted line represents the limit of detection (LOD). Error bars represent the standard error (*n* = 5). *P* < 0.05 (*), *P* < 0.0001 (****).

Immunohistochemistry (IHC) assays were used to observe the distribution of viral antigens in the testis and ductus deferens. IHC results showed that viral antigens were present in Sertoli cells, spermatogenic cells of various stages of the testis, and in the mucosal layer of the ductus deferens (Figure 3(a)). Viral RNA was then quantified by qRT‒PCR to assess the levels of viral replication in tissues. The results showed that the viral load in the ductus deferens was significantly higher than that in the testis at 4 dpi and that it was still higher at 8 dpi (Figure 3(b)).

### IBV infection affects the levels of plasma reproductive hormones and the transcription levels of corresponding hormone receptors

**Figure 4.**
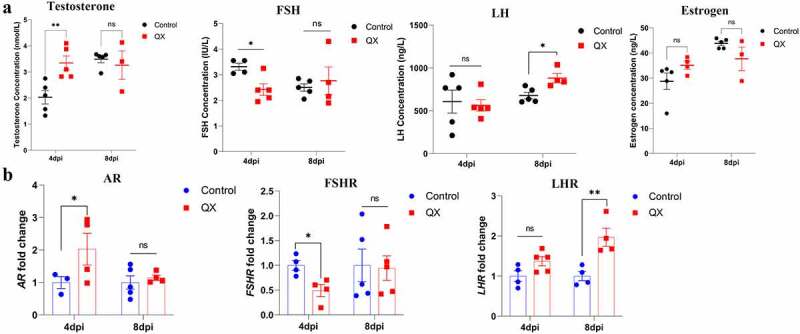
Effects of IBV infection on the concentrations of reproductive hormones in the plasma and the transcription levels of corresponding receptors in the testis. (a) Detection of the concentrations of testosterone (T), follicle-stimulating hormone (FSH), luteinizing hormone (LH) and estrogen (E2) in the plasma by ELISA. (b) Detection of the mRNA expression levels of androgen receptor (AR), follicle-stimulating hormone receptor (FSHR), and luteinizing hormone receptor (LHR) in the testis by qRT‒PCR. Values are expressed as the mean±sem (*n* = 5). *P* < 0.05 (*), *P* < 0.01 (**).

The reproductive physiology and behaviour of birds are governed by the endocrine system, and ELISA was therefore used to measure the concentrations of plasma reproductive hormones (T, FSH, LH, E2). A significant increase in plasma T concentration at 4 dpi was accompanied by a significant decrease in the FSH concentration in roosters (Figure 4(a)). At 8 dpi, only the LH concentration increased significantly (Figure 4(a)). Then, qRT‒PCR was used to detect the transcription levels of the corresponding receptors in the testis. Variation trend of hormone receptors was accordance with the hormones (Figure 4(b)).

### IBV infection alters the transcription levels of key enzymes involved in steroidogenesis

**Figure 5.**
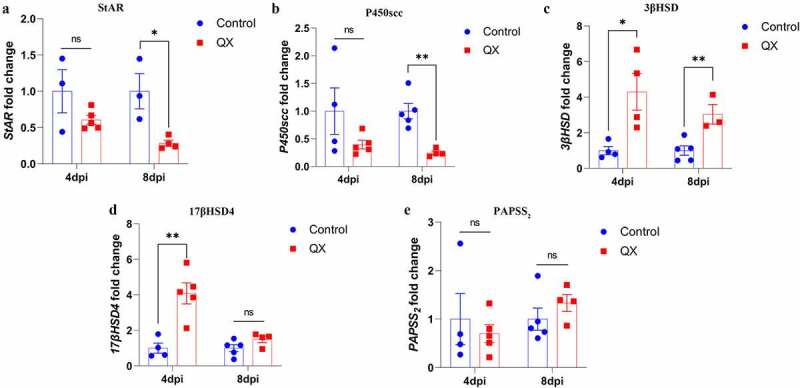
Effects of IBV infection on the mRNA expression levels of key enzymes involved in steroidogenesis. Values are expressed as the mean±sem (*n* = 5). *P* < 0.05 (*) and *P* < 0.01 (**).

To verify the plasma T concentration results, the transcription levels of StAR, P450scc, 3βHSD, 17βHSD4 and PAPSS_2_ in the testis were measured by qRT‒PCR. Compared with those in the control group, the transcription levels of 3βHSD and 17βHSD4 were significantly upregulated in the challenge group at 4 dpi (Figure 5(c,d)), whereas the transcription levels of StAR and P450scc were significantly decreased in the challenge group at 8 dpi (Figure 5(a,b)). These results suggest that IBV could affect the key enzymes to disturb the plasma T concentration.

### IBV induces extensive apoptosis of germ cells in the testis

**Figure 6.**
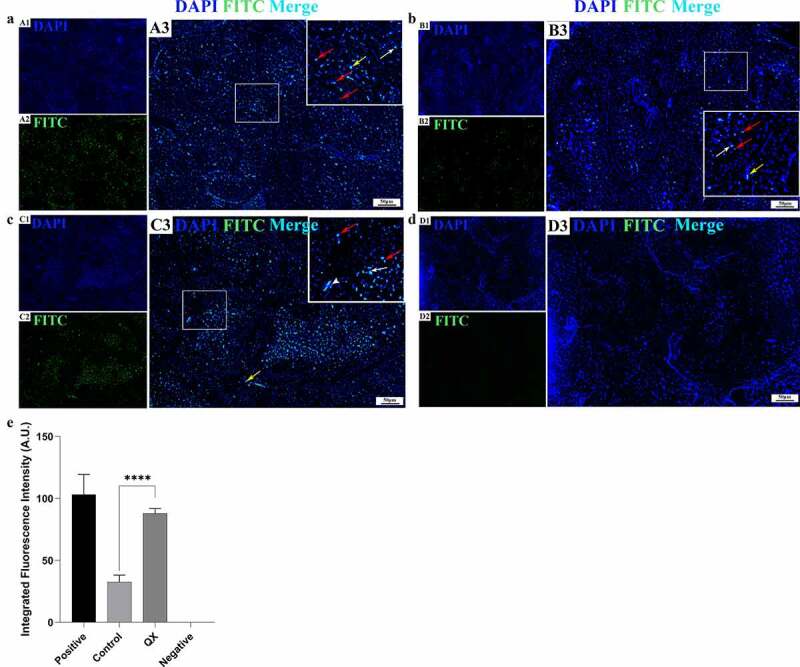
Detection of IBV-induced cellular apoptosis in the testis by TUNEL assay at 4 dpi. (a): Positive control section treated with a DNase I reaction mixture. (b): Control group. (c): QX group. (d): Negative control section treated without a TdT Enzyme reaction mixture. (**A1-D1**): The cell nuclei were stained with DAPI. (**A2-D2**): The apoptotic cells were observed via the green fluorescence of FITC. (**A3-D3**): Merge of blue and green fluorescence. Red arrows: spermatogenic cells; white arrows: Sertoli cells; white arrowhead: myoid cell; yellow arrows: Leydig cells. (**E**): The green fluorescence intensity representing the level of apoptosis. Error bars represent the standard error (*n* = 5). *P* < 0.0001 (****).

To assess whether IBV could induce apoptosis in the testis, a TUNEL assay was used to detect apoptosis at 4 dpi. The results showed only a limited number of apoptotic germ cells in the control group (Figure 6(B3)). However, in the challenge group, masses of apoptotic germ cells were observed (Figure 6(C3)), and the integrated fluorescence intensity was significantly higher than that of the control group (Figure 6(e)). These results indicate that IBV could induce extensive apoptosis of germ cells in the testis.

## Discussion

Several previous case reports have shown testicular atrophy in roosters due to in non-infectious causes and IBV infection [16]. The main non-infectious causes are feed management, such as poor environmental sanitation in poultry houses, an unbalanced feed ratio or a lack of certain trace elements, and ageing phenomena [27,28]. Cases involving IBV infection revealed marked atrophy of the testis, with congestion and hyperaemia at necropsy.

In this study, testicular morphological abnormalities and a significant reduction in testicular weight resulting from QX-type IBV infection were observed at necropsy. According to a previous report, the average weight of the left and right testis was≥2 g, but a testis with a weight<5 g can be judged as exhibiting moderate atrophy [28]. In addition, histopathological examination revealed dilatation of seminiferous tubules and a significant increase in their perimeter. These results demonstrated that QX-type IBV infection caused abnormal testicular morphology and moderate atrophy.

Innate immunity is the first line of defence against invading pathogens and plays an essential role in the early recognition and subsequent induction of a proinflammatory response [29]. In the present study, the transcription levels of proinflammatory cytokines and IFNs did not change significantly in the testis after viral infection, but the transcription level of OASL increased significantly. These results indicate that there is no inflammation or IFN response in the testis, which may be attributed to the strong protective effect of the blood-testis barrier. The significant upregulation of OASL transcription indicates that OASL may act as a host intrinsic restriction factor to directly inhibit viral replication. In the ductus deferens, many inflammatory cells and histopathological injuries were found, particularly in the mucous layer. The extent of the inflammatory response was also demonstrated by significantly upregulated transcription of proinflammatory cytokines and IFN-β. These results suggest that IBV causes more severe pathological injuries to the ductus deferens than to the testis, a difference that may be due to the strong blood-testicular barrier and the relatively weak barrier of the ductus deferens [30]. Notably, once the barriers are destroyed, spermatozoa and germ cells outside the barriers will be recognized by the body as foreign antigens, and the autoimmune response can be activated, such as in the case of autoimmune orchitis caused by mumps virus infection [31]. Therefore, although IBV did not cause obvious inflammation in the testis in this study, when spermatozoa were transported to the ductus deferens, they could also cause spermatozoan damage due to a strong inflammatory reaction, which leads to infertility.

In recent years, numerous studies have reported the extensive tissue tropism of IBV [32,33]. There are some differences among different genotypes, and some strains have high affinity for the reproductive system. Our previous study demonstrated that QX-type IBV can replicate in the different segments of the oviduct epithelium in laying hens [15]. In males, viral infection occurs in the spermatogonium and Sertoli cells and efferent ducts of the epididymis, and the virus carried in semen can be transmitted to hens by mating [18]. In this study, IHC results showed that QX-type IBV could replicate in Sertoli cells and germ cells at various stages and in the lamina propria of the ductus deferens. Further quantification of the virus showed that the viral load was significantly higher in the ductus deferens than in the testis. These results are consistent with the observation of more severe histopathologic changes in the ductus deferens, demonstrating a correlation between the viral replication level and tissue injury.

Spermatogenesis is regulated by the hypothalamic-pituitary-gonadal (testicular) axis [34], and LH secreted by the pituitary stimulates Leydig cells to secrete androgens [35]. FSH acts on Sertoli cell receptors to promote and regulate sperm production and maturation in the testis and is associated with the proliferation and differentiation of Sertoli cells [35]. In this study, increased T levels lead to immunosuppression in the testis, which plays an essential role in maintaining the blood-testis barrier [36], and this locally immunosuppressive anti-inflammatory environment may be the reason why IBV does not cause significant inflammation in the testis. The decreased FSH level causes abnormalities in Sertoli cells themselves and disrupts their function, which may lead to the development of testicular lesions. Therefore, aberrant reproductive hormone levels are also one of the important causes for testicular degeneration. Androgens bind to androgen-binding protein (ABP) in Sertoli cells to regulate spermatogenesis; however, whether ABP exists in birds is unknown [35]. Hormones need to bind to corresponding receptors in target cells to exert their effect, and the amounts of these receptors are in dynamic balance with the concentrations of hormones.

T synthesis proceeds from cholesterol that undergoes a series of enzymatic reactions in the Leydig cells of the testis. The first and rate-limiting step is the translocation of cholesterol from the outer to the inner mitochondrial membrane, which is promoted by StAR, and the conversion of cholesterol to pregnenolone is catalysed by P450scc in the inner mitochondrial membrane [37]. Pregnenolone, the common precursor of steroid hormones, is converted to 17-hydroxypregnenolone (17OH-pregnenolone) and then transformed into dehydroepiandrosterone (DHEA), which is catalysed by P450c17 [38]. DHEA, the precursor of active androgen, is converted to androstenedione by 3βHSD and then transformed into T by 17βHSD [38]. In this study, the results indicate that the virus-induced increase in plasma T concentration is due to the increased transcription of 3βHSD and 17βHSD4. Subsequently, the rate of T synthesis was significantly reduced by decreasing the transcription of StAR and P450scc, confirming the change in plasma T concentration.

As indicated earlier, IBV-caused injuries in the reproductive system and reproductive endocrine disruption may lead to the common endpoint of apoptosis. Previous studies have demonstrated that IBV can induce apoptosis in tracheal, kidney, and HD11 macrophages [39,40]. However, to the best of our knowledge, whether IBV can induce apoptosis in the testis is still unknown. In this study, we prove that IBV infection increases the level of apoptosis; whether the initial impacts are on the control mechanisms of the hypothalamic-pituitary-testicular axis or on Sertoli cells, spermatogenic cells and Leydig cells, the common endpoint is an increased number of apoptotic germ cells [41]. Excessive apoptosis is also a reflection of virus pathogenicity, which will cause irreversible injury to tissues and organs. As mentioned above, the moderate atrophy of the testis observed in this study may be directly due to extensive apoptosis. A previous study demonstrated that targeting apoptosis induced by virulent coronaviruses can reduce viral pathogenesis and disease severity [42]. In the future, the mechanism of IBV-induced apoptosis in the reproductive system should be further studied.

In addition, chickens of all ages are susceptible to IBV, and chicks less than one month old are especially susceptible. Whether infection early in life can also cause lesions in the reproductive system of roosters or even cause permanent injuries still needs to be systematically evaluated through further research.

## Conclusions

QX-type IBV replicates in the testis and ducts, causing severe damage, and induces endocrine disorders in reproductive organs and apoptosis of germ cells. These results provide a basic understanding of the pathogenicity of QX-type IBV in the male reproductive system and elucidate the possibility of IBV causing infertility in roosters via multiple pathways.

## Data Availability

All of the data extracted for this study are included in the manuscript.
